# Gypenoside Attenuates *β* Amyloid-Induced Inflammation in N9 Microglial Cells via SOCS1 Signaling

**DOI:** 10.1155/2016/6362707

**Published:** 2016-04-26

**Authors:** Hui Cai, Qianlei Liang, Guanqun Ge

**Affiliations:** ^1^Department of Vascular Surgery, The First Affiliated Hospital of Xi'an Jiaotong University, Xi'an 710061, China; ^2^The Second Department of Neurosurgery, China-Japan Union Hospital of Jilin University, Changchun 130033, China; ^3^Department of Breast Surgery, The First Affiliated Hospital of Xi'an Jiaotong University, Xi'an 710061, China

## Abstract

Reducing *β* amyloid- (A*β*-) induced microglial activation is believed to be effective in treating Alzheimer's disease (AD). Microglia can be activated into classic activated state (M1 state) or alternative activated state (M2 state), and the former is harmful; in contrast, the latter is beneficial. Gypenoside (GP) is the major bioactive constituent of* Gynostemma pentaphyllum*, a traditional Chinese herb medicine. In this study, we hypothesized that GP attenuates A*β*-induced microglial activation by ameliorating microglial M1/M2 states, and the process may be mediated by suppressor of cell signaling protein 1 (SOCS1). In this study, we found that A*β* exposure increased the levels of microglial M1 markers, including iNOS expression, tumor necrosis factor *α* (TNF-*α*), interleukin 1*β* (IL-1*β*), and IL-6 releases, and coadministration of GP reversed the increase of M1 markers and enhanced the levels of M2 markers, including arginase-1 (Arg-1) expression, IL-10, brain-derived neurotrophic factor (BDNF), and glial cell-derived neurotrophic factor (GDNF) releases in the A*β*-treated microglial cells. SOCS1-siRNA, however, significantly abolished the GP-induced effects on the levels of microglial M1 and M2 markers. These findings indicated that GP attenuates A*β*-induced microglial activation by ameliorating M1/M2 states, and the process may be mediated by SOCS1.

## 1. Introduction

Cerebral *β* amyloid (A*β*) deposition is a typical pathogenesis of Alzheimer's disease (AD). High-level of A*β* in brain can activate microglial cells to secrete proinflammatory factors, including tumor necrosis factor *α* (TNF-*α*), interleukin 1*β* (IL-1*β*), and IL-6, causing neural cell injury and even death [1–3]. Therefore, inhibiting A*β*-induced microglial activation is believed to be a useful therapy for AD. Some of the latest investigations showed that microglia can be activated into classic activated state (M1 state) or alternative/selective activated state (M2 state) [4]. M1 state of microglia can secrete high-level of proinflammatory factors, which are believed to be harmful; in contrast, M2 state of microglia may produce anti-inflammatory cytokines and neurotrophins, such as IL-10, brain-derived neurotrophic factor (BDNF), and glial cell-derived neurotrophic factor (GDNF); therefore M2 state of microglia is regarded to be beneficial [5–7]. Given these reasons above, switching M1 state of microglia to M2 state may be useful in treating microglial activation-induced neurological disorders.

*Gynostemma pentaphyllum* (*G. pentaphyllum*) is a traditional Chinese medicine, usually used as herb tea with a wide range of health benefits, including inhibition of inflammation and prevention of cardiovascular diseases [8, 9]. Some of the latest studies showed that gypenoside (GP) is the major bioactive constituent of* G. pentaphyllum* [10, 11]. In addition, a recent investigation reported that GP can induce neuroprotection against A*β* in vitro [12]. However, the exact neuroprotective mechanism of GP is still obscure.

Suppressor of cell signaling (SOCS) proteins are vital negative regulators of adaptive and innate immune responses [13, 14]. At present, at least eight structurally similar proteins have been discovered, including SOCS1–7 and the cytokine-inducible SH protein (CIS) [15–17]. It is reported that SOCS1 can induce the differentiation of macrophage from M1 to M2 state [18]. As microglial cells and macrophages are very similar in a variety of biological characters, such as activation and secretion of cytokines, microglial cells are regarded as the macrophages in central nervous system [19, 20].

In this study, we used N9 microglial cells exposed to A*β* to induce microglial activation and hypothesized that GP attenuates A*β*-induced microglial activation by ameliorating microglial M1/M2 states, and the process may be mediated by SOCS1.

## 2. Materials and Methods

### 2.1. Materials

N9 microglial cells were provided by the Department of Anesthesiology, Xijing Hospital, Fourth Military Medical University, Xi'an, China. The N9 microglial cells are very similar with the primary cultured microglial cells in being polarized and secreting inflammatory cytokines. Gypenoside was purchased from China National Pharmaceutical Group (Beijing, China) and dissolved in normal medium to treat cells. Betta amyloid1-42 (A*β*), Iscove's modified Dulbecco's medium (IMDM) and fetal bovine serum (FBS) were purchased from Sigma-Aldrich (St. Louis, USA). Anti-inducible nitric oxide synthase (iNOS) and anti-arginase-1 (Arg-1) primary antibodies were obtained from Chemicon (USA); anti-SOCS1 and anti-*β*-actin primary antibodies were purchased from Cell Signaling Technology, Inc. (USA); anti-ionized calcium binding adapter molecule 1 (IBA-1) primary antibody was purchased from Proteintech (USA); anti-GAPDH primary antibody, Cy-3 labeled secondary antibody, FITC-labeled secondary antibody, and DAPI staining solution were purchased from CWBIO (China). The enzyme-linked immunosorbent assay (ELISA) kits used in this study were purchased from PeproTech Inc. (USA).

### 2.2. Cell Culture

N9 microglial cells were cultured in the IMDM medium containing 5% FBS, 100 U/mL penicillin, 100 *μ*g/mL streptomycin, and 2 mM glutamine at 37°C, and the air of the incubator consisted of 5% CO_2_ and 95% air. The medium was changed every 2-3 days. The cells were passaged 2 times per week with a split ratio of 1 : 4.

### 2.3. Experimental Protocols

**Figure 1:**
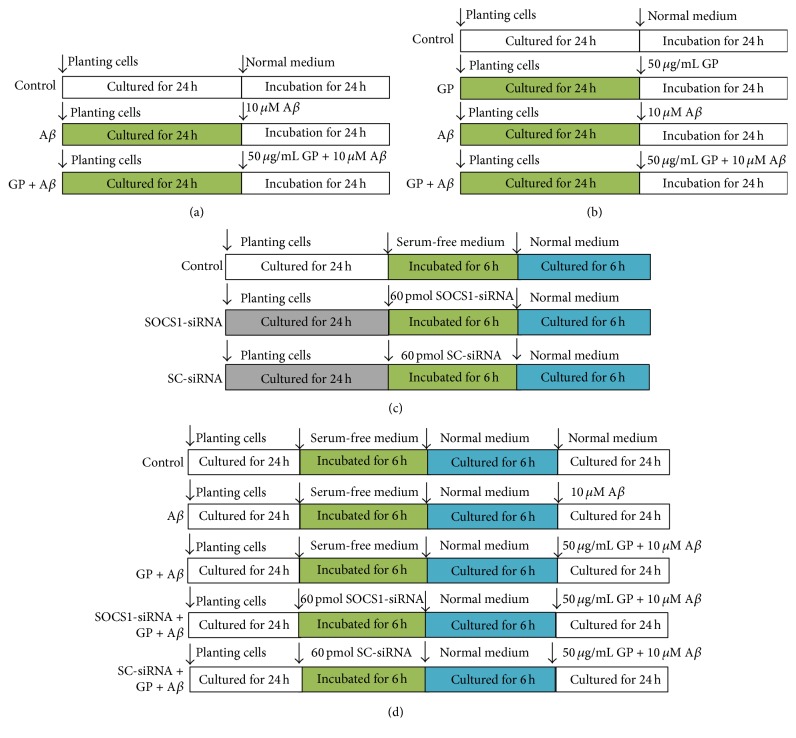
Experimental protocol diagram. (a) The N9 cells were divided into three groups; after being treated for 24 h as shown in the figure, western blot analysis and immunocytochemistry were performed to assess iNOS and Arg-1 protein expressions. (b) The cells were divided into four groups, after 24-h incubation, western blot and ELISA were used to evaluate IBA-1 expression and cytokines releases, respectively. (c) The cells were divided into three groups, after being treated as shown in the figure, western blot was used to evaluate the SOCS1-siRNA-induced SOCS1 protein expression. (d) The cells were divided into five groups, after the treatments, iNOS and Arg-1 expressions and cytokines releases were measured.

The cells were divided into three groups, including control, A*β*, and GP + A*β* groups; after being treated for 24 h, western blot and immunocytochemistry were used to evaluate iNOS and Arg-1 expressions (Figure 1(a)). Then, to observe microglial activation marker IBA-1 expression and cytokines releases, the cells were divided into four groups, including control, GP alone, A*β*, and GP + A*β* groups; after being treated for 24 h, western blot was taken to assess IBA-1 expression, and ELISA kits were used to measure the levels of cytokines in the medium (Figure 1(b)). To evaluate the SOCS1-siRNA-induced downregulation degree of SOCS1 protein, the cells were divided into three groups, including control, SOCS1-siRNA, and scrambled (SC)-siRNA groups; after being incubated in the serum-free medium or the serum-free medium containing 60 pmol siRNA for 6 h, the medium was replaced with normal medium; after an additional 6-h incubation, western blot was used to evaluated SOCS1 protein expression (Figure 1(c)). Then, we investigated the role of SOCS1 in the GP-induced effects on microglial activation. The cells were divided into five groups, including control, A*β*, GP + A*β*, SOCS1-siRNA + GP + A*β*, and SC-siRNA + GP + A*β* groups; after the treatments (Figure 1(d)), western blot was taken to evaluate iNOS and Arg-1 expressions, and ELISA kits were taken to measure the cytokine levels in the medium.

### 2.4. Western Blot Analysis

The N9 microglial cells were seeded into a six-well cell culture plate at a density of 2 × 10^5^ cells/well. After the treatments, the cells were collected, and the total protein was evaluated by using the Bradford method. The western blot analysis was performed as previously described [21]. The following primary antibodies were used in this investigation, including anti-iNOS (1 : 1000), anti-Arg-1 (1 : 1000), anti-IBA-1 (1 : 1000), anti-SOCS1 (1 : 1000), anti-GAPDH (1 : 1000), and anti-*β*-actin (1 : 1000). Chemiluminescence technique (Amersham Pharmacia Biotech Piscataway, USA) was used to detect the antigens. The experiments were repeated four times (*n* = 4) for each protein expression. Image analysis was performed by using computerized analysis software (Bio-Rad Laboratories, Hercules, USA).

### 2.5. Immunocytochemistry

The cells were seeded into confocal microscopy special dishes at a density of 1 × 10^4^ cells per dish. After the incubations, the treated microglial cells were fixed with 4% paraformaldehyde solution for 30 min. The cells were then blocked with 5% BSA for 15 min. The cells were incubated at 4°C overnight with primary antibody (iNOS, 1 : 100; Arg-1, 1 : 100). After the incubation, the cells were exposed to Cy3-labeled (red) or FITC-labeled (green) antibody solution (1 : 200) for 1 h at room temperature. At the end of the exposure, 200 *μ*L of DAPI staining solution was added into the dish. After 10-min incubation, the dish was washed three times with PBS. Then the dish was observed by using a confocal microscope (Olympus, Japan). The observations were performed in three independent experiments, and the images were taken randomly.

### 2.6. Enzyme-Linked Immunosorbent Assay

The cells were seeded into a 96-well plate at a density of 1 × 10^5^ cells per well, and six wells per group (*n* = 6). The cytokine concentration in the supernatant was evaluated by using the corresponding ELISA kit. The supernatants were harvested and measured for cytokine concentrations, including TNF-*α*, IL-1*β*, IL-6, IL-10, BDNF, and GDNF by using the corresponding ELISA kit, according to the manufacturer's instructions. And the results are expressed as picograms per milliliter.

### 2.7. SOCS1 mRNA Silencing

The SOCS1-siRNA and scrambled-siRNA (SC-siRNA) were purchased from Santa Cruz Biotechnology (USA). The cells were transfected with 60 pmol SOCS1-siRNA using the Lipofectamine reagent (Invitrogen, USA) in serum-free medium according to the manufacturer's instructions. The cells were incubated for 6 h and recovered for an additional 6 h before the treatments. The experiments were repeated four times (*n* = 4). The SC-siRNA served as the negative control.

### 2.8. Statistical Analysis

SPSS13.0 for Windows (SPSS Inc., USA) was used to conduct statistical analysis. Values are expressed as means ± standard deviation (SD). Results were compared by one-way ANOVA, followed by Tukey's Multiple Comparison Test. *P* < 0.05 indicates statistical significance.

## 3. Results

### 3.1. GP Attenuated iNOS Protein Expression in A*β*-Treated Microglial Cells Dose-Dependently

**Figure 2:**
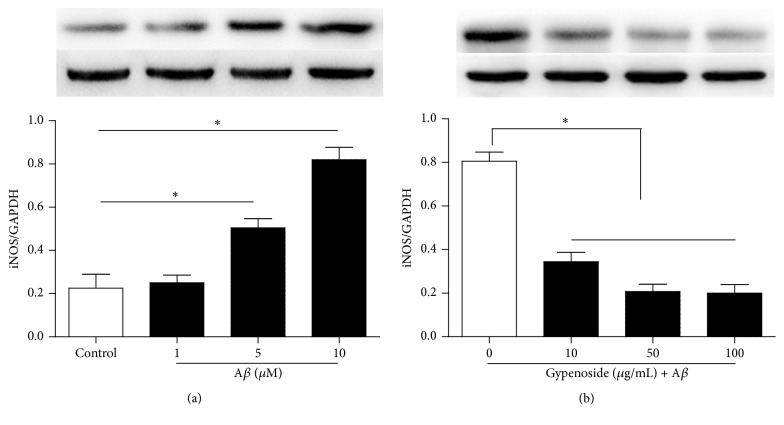
GP attenuated iNOS protein expression in A*β*-stimulated microglial cells in a dose-dependent manner. The microglial cells were exposed to different concentrations of A*β* for 24 h; then the iNOS expression was assessed by using western blot (a). The cells were exposed to different concentrations of GP in the presence of 10 *μ*M A*β* for 24 h; then the iNOS expression was assessed (b). Data are means ± SD, *n* = 4, *∗*: *P* < 0.05.

To determine a suitable A*β* concentration, the N9 microglial cells were exposed to 1 *μ*M, 5 *μ*M, and 10 *μ*M A*β*, respectively. After 24-h incubation, the iNOS protein expression, a marker of microglial M1 state, was assessed by using western blot. Compared with the control cells, 5 *μ*M and 10 *μ*M A*β* increased iNOS protein expression significantly (*P* < 0.05), and 10 *μ*M A*β* was taken in the subsequent experiments (Figure 2(a)).

Then, microglial cells were exposed to 10 *μ*g/mL, 50 *μ*g/mL, and 100 *μ*g/mL GP in the presence of 10 *μ*M A*β* for 24 h. Compared with the cells treated with 10 *μ*M A*β* alone, 10 *μ*g/mL, 50 *μ*g/mL, and 100 *μ*g/mL GP decreased iNOS expression obviously (*P* < 0.05), and 50 *μ*g/mL GP was used in the subsequent experiments (Figure 2(b)).

### 3.2. GP Decreased iNOS and Increased Arg-1 Expressions in Microglial Cells Exposed to A*β*

**Figure 3:**
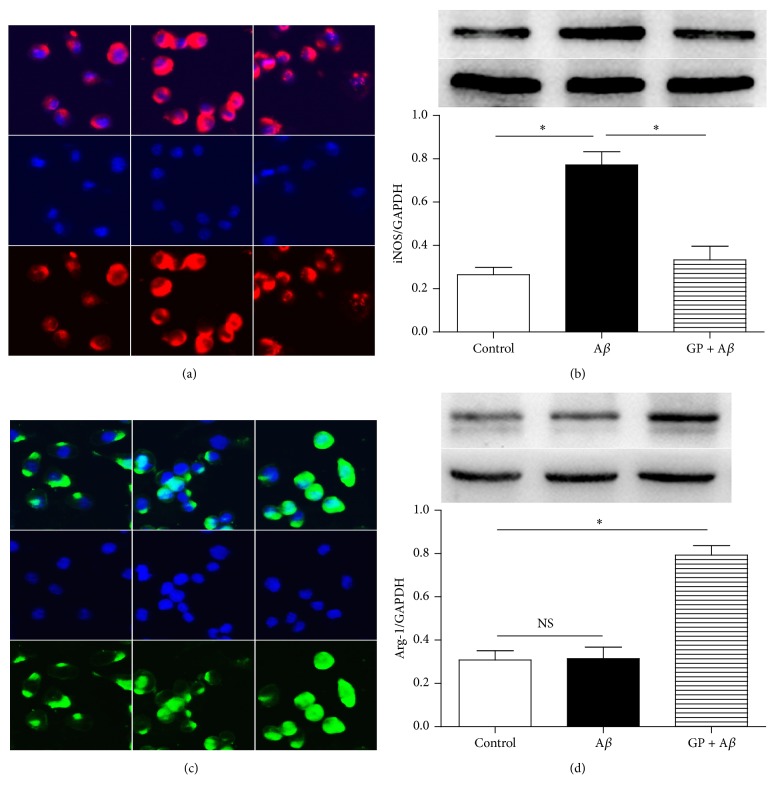
GP abolished A*β*-induced effects on iNOS and Arg-1 expressions. The N9 microglial cells were divided into three groups, including control: cells cultured in normal medium; A*β*: cells cultured in the medium containing 10 *μ*M A*β*; GP + A*β*: cells cultured in the medium containing 50 *μ*g/mL GP and 10 *μ*M A*β*. After 24-h incubation, western blot (*n* = 4) and immunocytochemistry were taken to determine the iNOS (a-b) and Arg-1 (c-d) expressions. Data are means ± SD, *∗*: *P* < 0.05, NS: no significance.

To observe the GP-induced effects on microglial M1 state marker iNOS and M2 state marker Arg-1 expressions, western blot and immunocytochemistry were used. Compared with the control, 10 *μ*M A*β* increased the expression of iNOS protein significantly (*P* < 0.05); meanwhile, coadministration of 50 *μ*g/mL GP partially reversed the A*β*-induced upregulation of iNOS protein (Figures 3(a) and 3(b)) and increased Arg-1 expression significantly (Figures 3(c) and 3(d), *P* < 0.05). These findings indicated that GP exposure ameliorates the biomarker levels of microglial M1 and M2 states.

### 3.3. GP Decreased IBA-1 Protein Expression and Ameliorated the Cytokine Levels of Microglial M1 and M2 States

**Figure 4:**
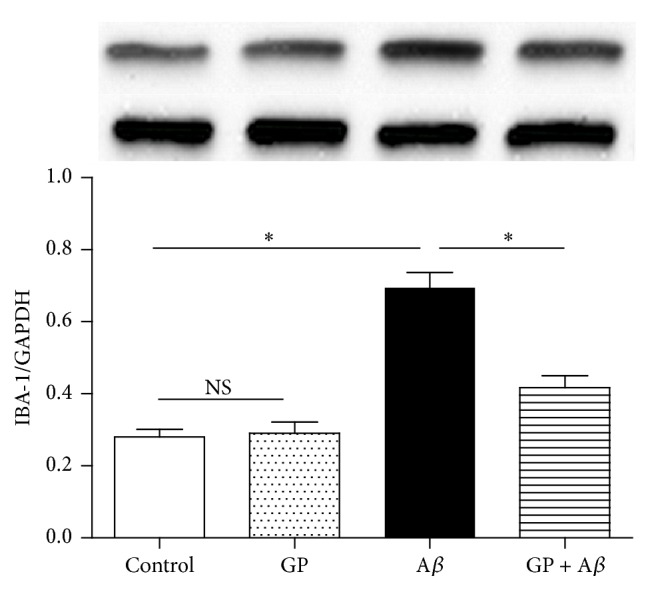
GP partially abolished A*β*-induced effects on IBA-1 protein expression. The N9 microglial cells were divided into four groups, including control: cells cultured in normal medium; GP: cells cultured in the medium containing 50 *μ*g/mL GP; A*β*: cells cultured in the medium containing 10 *μ*M A*β*; GP + A*β*: cells cultured in the medium containing 50 *μ*g/mL GP and 10 *μ*M A*β*. After 24-h incubation, western blot (*n* = 4) was taken to measure the IBA-1 expression. Data are means ± SD, *∗*: *P* < 0.05, NS: no significance.

Western blot was taken to evaluate the IBA-1 protein expression, which could reflect microglial activation level. Compared with the control, GP alone did not induce a significant effect on IBA-1 expression (*P* > 0.05), and A*β* of 10 *μ*M increased IBA-1 expression significantly (*P* < 0.05); however, GP of 50 *μ*g/mL partially abolished the A*β*-induced upregulation of IBA-1 expression (*P* < 0.05, Figure 4).

**Figure 5:**
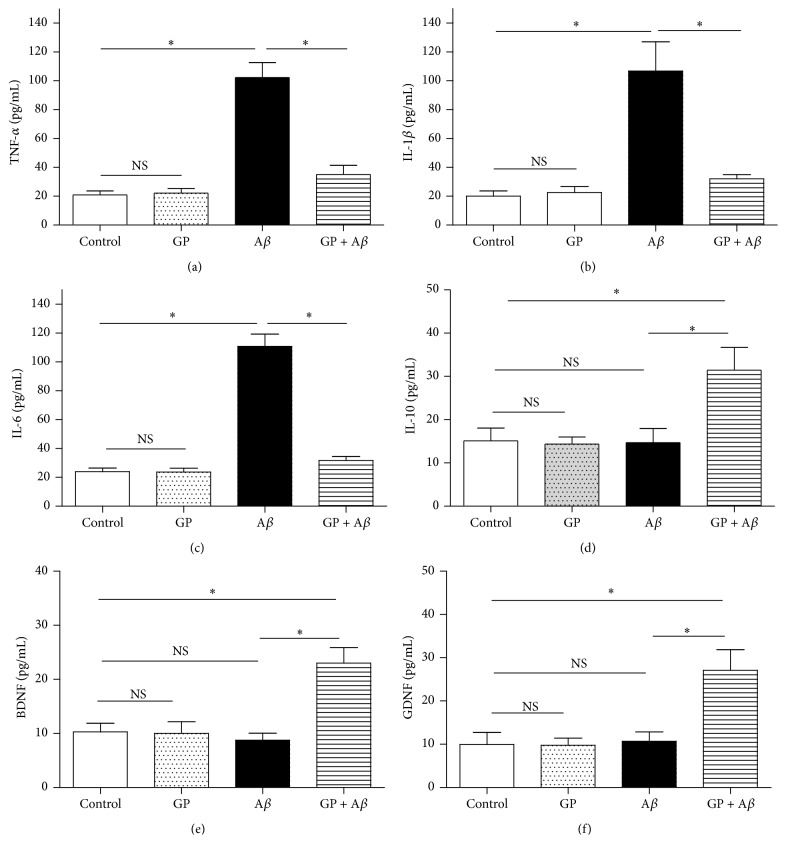
GP reversed A*β*-induced effects on the releases of cytokines. The N9 microglial cells were divided into four groups, including control: cells cultured in normal medium; GP: cells cultured in the medium containing 50 *μ*g/mL GP; A*β*: cells cultured in the medium containing 10 *μ*M A*β*; GP + A*β*: cells cultured in the medium containing 50 *μ*g/mL GP and 10 *μ*M A*β*. After 24-h incubation, the corresponding ELISA kits were used to evaluate the releases of cytokines, including TNF-*α* (a), IL-1*β* (b), IL-6 (c), IL-10 (d), BDNF (e), and GDNF (f). Data are means ± SD, *n* = 6, *∗*: *P* < 0.05, NS: no significance.

The corresponding ELISA kits were taken to measure cytokine levels in the medium. Compared with the control, A*β* of 10 *μ*M increased the TNF-*α*, IL-1*β*, and IL-6 concentrations (*P* < 0.05) and did not induce obvious effects on IL-10, BDNF, and GDNF concentrations in the medium (*P* > 0.05) after a 24-h incubation; GP of 50 *μ*g/mL, however, significantly decreased TNF-*α*, IL-1*β*, and IL-6 concentrations (Figures 5(a)–5(c)) and, meanwhile, increased the levels of IL-10, BDNF, and GDNF concentrations markedly (*P* < 0.05, Figures 5(d)–5(f)). These findings indicated that GP exposure decreased the markers of microglial M1 state and increased that of M2 state in the A*β*-treated N9 microglial cells.

### 3.4. SOCS1-siRNA Reversed the GP-Induced Effects on Microglial M1 and M2 Markers

**Figure 6:**
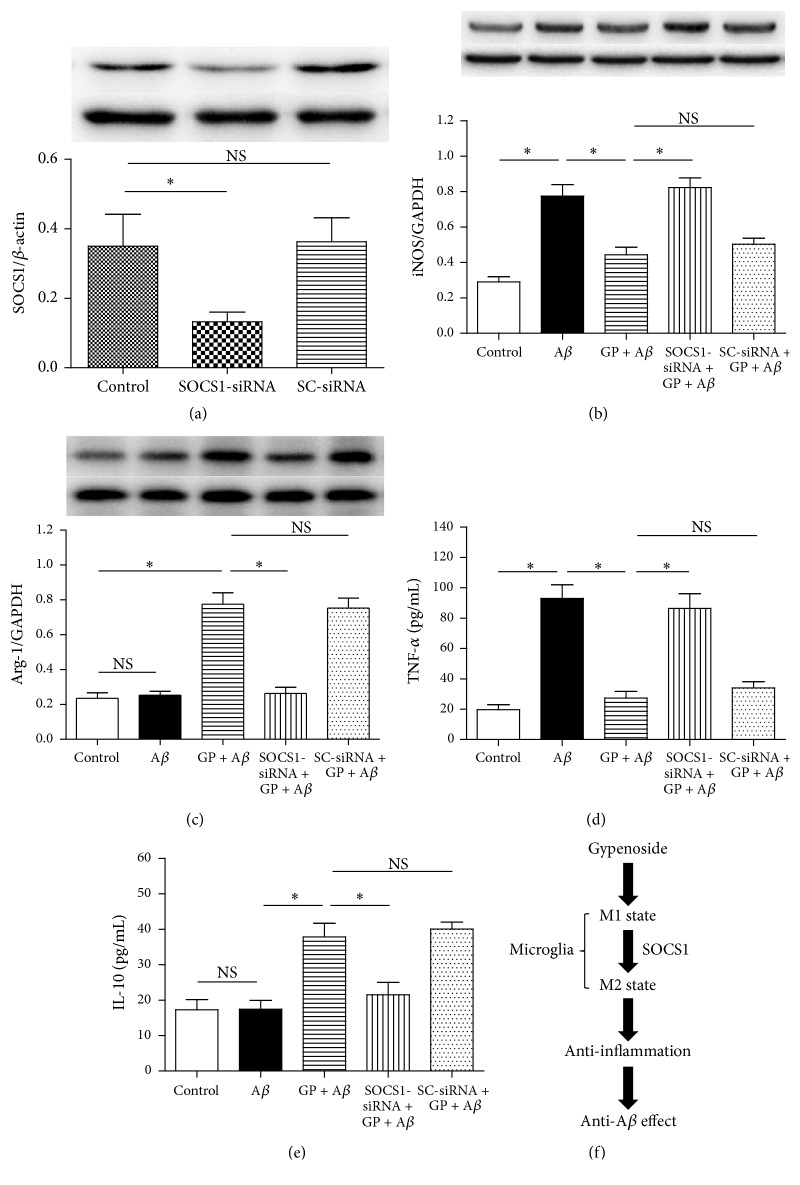
SOCS1-siRNA abolished GP-induced effects on iNOS and Arg-1 expressions and TNF-*α* and IL-10 releases. Western blot was taken to evaluate the SOCS1-siRNA-induced effect on SOCS1 protein expression ((a), *n* = 4). Then the cells were divided into five groups, including control: cells cultured in normal medium; A*β*: cells cultured in the medium containing 10 *μ*M A*β*; GP + A*β*: cells cultured in the medium containing 50 *μ*g/mL GP and 10 *μ*M A*β*; SOCS1-siRNA + GP + A*β*: cells treated with SOCS1-siRNA for 6 h and then exposed to the medium containing 50 *μ*g/mL GP and 10 *μ*M A*β*; SC-siRNA + GP + A*β*: cells treated with scrambled (SC)-siRNA for 6 h and then exposed to the medium containing 50 *μ*g/mL GP and 10 *μ*M A*β*. After 24-h incubation, the iNOS and Arg-1 expressions were evaluated by using western blot analysis ((b)-(c), *n* = 4), and TNF-*α* and IL-10 concentrations were assessed by using ELISA kits ((d)-(e), *n* = 6). (f) Hypothetical model of SOCS1 mediates GP-induced anti-A*β* effect by switching microglia from M1 to M2 state. Data are means ± SD, *∗*: *P* < 0.05, NS: no significance.

To observe the role of SOCS1 protein in GP-induced effects on A*β*-treated microglia, we used SOCS1-siRNA. Compared with the control (Figure 6(a)), SOCS1-siRNA downregulated SOCS1 protein expression significantly (*P* < 0.05), and SC-siRNA did not induce significant effect on it (*P* > 0.05), indicating that the SOCS1-siRNA was effective in inhibiting the SOCS1 protein expression in N9 microglial cells.

Then, we observed SOCS1-siRNA-induced effects on the markers of microglial M1 and M2 states. Compared with the cells treated with 50 *μ*g/mL GP plus 10 *μ*M A*β*, the SOCS1-siRNA reversed the GP-induced effects on iNOS and Arg-1 protein expressions (Figures 6(b) and 6(c)), and the releases of TNF-*α* and IL-10 (Figures 6(d) and 6(e)) and SC-siRNA did not induce significant effects on the protein expressions and cytokine releases. These findings indicated that SOCS1 may mediate GP-induced microglial phenotypic transformation (Figure 6(f)).

## 4. Discussion

In this study, we found that GP attenuated A*β*-induced microglial activation, decreased the levels of microglial M1 state markers, including iNOS protein expression, TNF-*α*, IL-1*β*, and IL-6 releases, and increased the levels of M2 markers, such as Arg-1 protein expression, IL-10, BDNF, and GDNF secretions from the cells. SOCS1-siRNA, but not SC-siRNA, partially abolished these GP-induced effects above. These results indicated that GP reduces the A*β*-induced microglial activation by shifting microglial M1 to M2 state, and the SOCS1 protein may mediate the process.

Cerebral A*β* deposition is one of the most typical pathological characters in patients with AD [22]. High-level of A*β* in the diseased brain can stimulate microglia and activate them to secrete proinflammatory factors, including TNF-*α*, IL-1*β*, and IL-6, resulting in inflammatory brain injury [1–3]. Therefore, inhibiting A*β*-induced microglial activation is regarded as an effective therapy for AD [23]. Some of the latest investigations showed that microglia can be activated into classic activated state (M1 state) or alternative/selective activated state (M2 state) [4]. In this study, we used A*β*1–42 to activate N9 microglia into M1 state, causing elevated IBA-1 and iNOS protein expressions and TNF-*α*, IL-1*β*, and IL-6 releases from the cells.* G. pentaphyllum* is a traditional Chinese herb medicine, and it is a perennial liana indigenous to Southern China, Japan, Korea, and India [24]. Some investigations showed that* G. pentaphyllum* exhibits a variety of biological functions, such as immunopotentiating, cholesterol-lowering, antitumor, antioxidative, anti-inflammatory, antihyperlipidemic, and antihypoglycemic effects [9, 25–27]. GP has been identified to be the main bioactive substance of* G. pentaphyllum* [10, 11]. Although some studies indicated that GP could reduce the activation of macrophage [27], at present, it has not been reported whether GP can reduce A*β*-induced microglial activation. In this study, we observed that GP reduced the release of proinflammatory factors and increased anti-inflammatory and neurotrophic factor releases, indicating that GP attenuates microglial activation by ameliorating microglial M1/M2 states. Similarly, in a study related to macrophage, researchers found that GP attenuated lipopolysaccharide- (LPS-) induced macrophage activation [28], as microglia are very similar with macrophages in producing cytokines and activation [19, 20], which are considered to be the macrophages in the central nervous system. In addition, the N9 microglia is a murine microglia cell line, which can be activated into M1 or M2 state and release cytokines in the presence of stimulus; therefore these characters of N9 microglia cell line are very similar to those of the primary cultured microglia [29]. For this reason, we used N9 microglia in the current investigation.

The suppressors of cytokine signaling (SOCS) are proteins that restrict the functions of cytokines [13, 14]. At present, at least eight SOCS proteins have been found, including SOCS1–7 and cytokine-inducible SH2 protein (CIS) [15–17]. All the SOCS members share a central SRC-homology 2 (SH2) domain, a variable N-terminal region containing an extended SH2 subdomain (ESS), and a conserved SOCS box at the C-terminus [13]. Among the SOCS proteins family there are closer structural relationships in pairs: CIS and SOCS2, SOCS1 and 3, SOCS4 and 5, and SOCS6 and 7. Unlike other SOCS proteins, SOCS1 and SOCS3 are expressed in microglia, and some studies showed that SOCS1, but not SOCS3, can induce the differentiation of microglial M2 state [13]. Therefore, we investigated the role of SOCS1 protein in this study. We observed that SOCS1-siRNA significantly abolished the GP-induced effects on iNOS and Arg-1 protein expressions and the releases of cytokines, indicating that SOCS1 protein may mediate the GP-induced modulation of microglial M1/M2 states in the A*β*-treated N9 microglial cells. Similarly, Dragone et al. found that resveratrol inhibits LPS-induced microglial activation via modulating SOCS1 signaling [30]. And Cardoso et al. also reported that SOCS1 is involved in miR-155-induced anti-inflammatory effects in microglial cells [31]. Our and the other researchers' findings indicated that SOCS1 is really a key target modulating microglial activated state. In addition, Meng et al. found that GP attenuates A*β*-induced neurotoxicity in differentiated PC12 cells via PI3K/Akt pathway [12]. Choi et al. found that GP protected neurons in substantia nigra and recovered the level of dopamine in rat brain [32]; these findings indicated that GP may prevent or treat other neurological disorders, such as Parkinson's disease.

In this investigation, we found that GP attenuates A*β*-induced microglial activation via SOCS1 signaling. Our findings indicated that GP may be an effective drug in reducing the neuroinflammation of AD or even treat AD, and SOCS1 protein can be a potential therapeutic target for the treatment of microglial activation-induced neuroinflammatory disorders. However, there are still some limitations in our study. First, the GP used in this study is made up of not just one molecule; in fact, more than 90 dammarane-type saponin glycosides (called gypenosides) have been identified phytochemically by gas chromatography-mass spectrometry [27], what more vital role GP molecule played in this study is still obscure. Second, this study is just performed in N9 microglia cell line; therefore our findings should be verified in primary cultured microglial cells and in vivo.

In summary, this study has showed that GP attenuates A*β*-induced microglial activation by ameliorating microglial M1/M2 states, and SOCS1 may mediate the process.

## References

[1] Villemagne V. L., Burnham S., Bourgeat P. (2013). Amyloid *β* deposition, neurodegeneration, and cognitive decline in sporadic Alzheimer's disease: a prospective cohort study. *The Lancet Neurology*.

[2] Liu H., Wang J., Wang J., Wang P., Xue Y. (2015). Paeoniflorin attenuates A*β*1–42-induced inflammation and chemotaxis of microglia in vitro and inhibits NF-*κ*B- and VEGF/Flt-1 signaling pathways. *Brain Research*.

[3] Hong H. S., Maezawa I., Petrlova J. (2015). Tomoregulin (TMEFF2) binds Alzheimer's disease amyloid-*β* (A*β*) oligomer and A*β*PP and protects neurons from A*β*-induced toxicity. *Journal of Alzheimer's Disease*.

[4] Biswas S. K., Chittezhath M., Shalova I. N., Lim J.-Y. (2012). Macrophage polarization and plasticity in health and disease. *Immunologic Research*.

[5] Du C., Jin M., Hong Y. (2014). Downregulation of cystathionine *β*-synthase/hydrogen sulfide contributes to rotenone-induced microglia polarization toward M1 type. *Biochemical and Biophysical Research Communications*.

[6] Zhou X., He X. J., Ren Y. (2014). Function of microglia and macrophages in secondary damage after spinal cord injury. *Neural Regeneration Research*.

[7] Habib P., Slowik A., Zendedel A., Johann S., Dang J., Beyer C. (2014). Regulation of hypoxia-induced inflammatory responses and M1-M2 phenotype switch of primary rat microglia by Sex steroids. *Journal of Molecular Neuroscience*.

[8] He Q., Li J.-K., Li F. (2015). Mechanism of action of gypenosides on type 2 diabetes and non-alcoholic fatty liver disease in rats. *World Journal of Gastroenterology*.

[9] Lin J. M., Lin C. C., Chiu H. F., Yang J. J., Lee S. G. (1993). Evaluation of the anti-inflammatory and liver-protective effects of *Anoectochilus formosanus*, *Ganoderma lucidum* and *Gynostemma pentaphyllum* in rats. *The American Journal of Chinese Medicine*.

[10] Zhang L., Lin Y., Guan H., Hu L., Pan G. (2015). Simultaneous determination of gypenoside LVI, gypenoside XLVI, 2*α*-OH-protopanaxadiol and their two metabolites in rat plasma by LC-MS/MS and its application to pharmacokinetic studies. *Journal of Chromatography B: Analytical Technologies in the Biomedical and Life Sciences*.

[11] Shi L., Pi Y., Luo C., Zhang C., Tan D., Meng X. (2015). In vitro inhibitory activities of six gypenosides on human liver cancer cell line HepG2 and possible role of HIF-1*α* pathway in them. *Chemico-Biological Interactions*.

[12] Meng X., Wang M., Sun G. (2014). Attenuation of A*β*25-35-induced parallel autophagic and apoptotic cell death by gypenoside XVII through the estrogen receptor-dependent activation of Nrf2/ARE pathways. *Toxicology and Applied Pharmacology*.

[13] Łabuzek K., Suchy D., Gabryel B., Pierzchała O., Okopień B. (2012). Role of the SOCS in monocytes/macrophages-related pathologies. Are we getting closer to a new pharmacological target?. *Pharmacological Reports*.

[14] Dhawan R., Gupta K., Kajla M. (2015). Molecular characterization of SOCS gene and its expression analysis on *Plasmodium berghei* infection in *Anopheles culicifacies*. *Acta Tropica*.

[15] Masuhara M., Sakamoto H., Matsumoto A. (1997). Cloning and characterization of novel CIS family genes. *Biochemical and Biophysical Research Communications*.

[16] Piessevaux J., Lavens D., Peelman F., Tavernier J. (2008). The many faces of the SOCS box. *Cytokine & Growth Factor Reviews*.

[17] Starr R., Hilton D. J. (1998). SOCS: suppressors of cytokine signalling. *The International Journal of Biochemistry & Cell Biology*.

[18] Whyte C. S., Bishop E. T., Rückerl D. (2011). Suppressor of cytokine signaling (SOCS)1 is a key determinant of differential macrophage activation and function. *Journal of Leukocyte Biology*.

[19] Won S., Lee J.-K., Stein D. G. (2015). Recombinant tissue plasminogen activator promotes, and progesterone attenuates, microglia/macrophage M1 polarization and recruitment of microglia after MCAO stroke in rats. *Brain, Behavior, and Immunity*.

[20] Suenaga J., Hu X., Pu H. (2015). White matter injury and microglia/macrophage polarization are strongly linked with age-related long-term deficits in neurological function after stroke. *Experimental Neurology*.

[21] Bénard G., Massa F., Puente N. (2012). Mitochondrial CB_1_ receptors regulate neuronal energy metabolism. *Nature Neuroscience*.

[22] Wu Z., Zhao L., Chen X., Cheng X., Zhang Y. (2015). Galantamine attenuates amyloid-*β* deposition and astrocyte activation in APP/PS1 transgenic mice. *Experimental Gerontology*.

[23] Guo H. B., Cheng Y. F., Wu J. G. (2015). Donepezil improves learning and memory deficits in APP/PS1 mice by inhibition of microglial activation. *Neuroscience*.

[24] Cui J.-F., Eneroth P., Bruhn J. G. (1999). cynostemma pentaphyllum: identification of major sapogenins and differentiation from *Panax* species. *European Journal of Pharmaceutical Sciences*.

[25] Li L., Jiao L.-P., Lau B. H. S. (1993). Protective effect of gypenosides against oxidative stress in phagocytes, vascular endothelial cells and liver microsomes. *Cancer Biotherapy*.

[26] la Cour B., Mølgaard P., Yi Z. (1995). Traditional Chinese medicine in treatment of hyperlipidaemia. *Journal of Ethnopharmacology*.

[27] Aktan F., Henness S., Roufogalis B. D., Ammit A. J. (2003). Gypenosides derived from *Gynostemma pentaphyllum* suppress NO synthesis in murine macrophages by inhibiting iNOS enzymatic activity and attenuating NF-*κ*B-mediated iNOS protein expression. *Nitric Oxide*.

[28] Huang T. H.-W., Li Y., Razmovski-Naumovski V. (2006). Gypenoside XLIX isolated from *Gynostemma pentaphyllum* inhibits nuclear factor-kappaB activation via a PPAR-alpha-dependent pathway. *Journal of Biomedical Science*.

[29] Ma L., Jia J., Liu X., Bai F., Wang Q., Xiong L. (2015). Activation of murine microglial N9 cells is attenuated through cannabinoid receptor CB2 signaling. *Biochemical and Biophysical Research Communications*.

[30] Dragone T., Cianciulli A., Calvello R., Porro C., Trotta T., Panaro M. A. (2014). Resveratrol counteracts lipopolysaccharide-mediated microglial inflammation by modulating a SOCS-1 dependent signaling pathway. *Toxicology in Vitro*.

[31] Cardoso A. L., Guedes J. R., Pereira de Almeida L., Pedroso de Lima M. C. (2012). miR-155 modulates microglia-mediated immune response by down-regulating SOCS-1 and promoting cytokine and nitric oxide production. *Immunology*.

[32] Choi H. S., Park M. S., Kim S. H., Hwang B. Y., Lee C. K., Lee M. K. (2010). Neuroprotective effects of herbal ethanol extracts from *Gynostemma pentaphyllum* in the 6-hydroxydopamine-lesioned rat model of Parkinson's disease. *Molecules*.

